# Neurophenomenology of induced and natural synaesthesia

**DOI:** 10.1098/rstb.2019.0030

**Published:** 2019-10-21

**Authors:** David J. Schwartzman, Daniel Bor, Nicolas Rothen, Anil K. Seth

**Affiliations:** 1 Sackler Centre for Consciousness Science, University of Sussex, Brighton BN1 9QJ, UK; 2 Department of Informatics, University of Sussex, Brighton BN1 9QJ, UK; 3 Azrieli Programme on Brain, Mind, and Consciousness, Canadian Institute for Advanced Research, Toronto, Ontario, Canada; 4 Department of Psychology, University of Cambridge, Downing Street, Cambridge CB2 3EB, UK; 5 Faculty of Psychology, Swiss Distance University Institute, 3900 Brig, Switzerland

**Keywords:** synaesthesia, training, plasticity, perception, phenomenology, consciousness

## Abstract

People with synaesthesia have additional perceptual experiences, which are automatically and consistently triggered by specific inducing stimuli. Synaesthesia therefore offers a unique window into the neurocognitive mechanisms underlying conscious perception. A long-standing question in synaesthesia research is whether it is possible to artificially induce non-synaesthetic individuals to have synaesthesia-like experiences. Although synaesthesia is widely considered a congenital condition, increasing evidence points to the potential of a variety of approaches to induce synaesthesia-like experiences, even in adulthood. Here, we summarize a range of methods for artificially inducing synaesthesia-like experiences, comparing the resulting experiences to the key hallmarks of natural synaesthesia which include consistency, automaticity and a lack of ‘perceptual presence’. We conclude that a number of aspects of synaesthesia can be artificially induced in non-synaesthetes. These data suggest the involvement of developmental and/or learning components in the acquisition of synaesthesia, and they extend previous reports of perceptual plasticity leading to dramatic changes in perceptual phenomenology in adults.

This article is part of a discussion meeting issue ‘Bridging senses: novel insights from synaesthesia’.

## 1. Introduction

Synaesthesia is defined by the presence of additional perceptual experiences, which are automatically and consistently triggered by specific inducing stimuli [1]. For example, in grapheme-colour synaesthesia, the letter ‘A’ printed in black (inducer) may elicit a red colour experience (concurrent). Within cognitive neuroscience, synaesthesia is fascinating for a number of reasons. First, whether in grapheme-colour form or in any of its numerous other forms, it provides insight into the range and flexibility of perceptual experiences. The perceptually vivid nature of synaesthesia may also help unlock clues about the neurobiology of perceptual consciousness. Finally, given that synaesthesia often confers performance advantages for synaesthetic stimuli [2,3], studying synaesthesia may engender a deeper understanding of developmental learning processes.

Although synaesthesia was originally considered a condition one was born with [4], or at least one that was fixed from early childhood [5,6], increasing evidence points to the possibility of inducing hallmarks of synaesthetic experience in adulthood, through a variety of methods including training, hypnosis and various other means [7–12]. This emerging research sheds new light on the potential plasticity of the adult brain to develop strikingly novel perceptual phenomenology. Therefore, artificially inducing synaesthesia may offer a powerful experimental suite of protocols to richly explore perception and cognition. In this paper, we summarize this emerging body of synaesthesia induction research, reviewing examples of artificially induced synaesthesia and examining its implications for learning and perceptual plasticity.

## 2. Natural synaesthesia

The defining hallmarks of genuine, natural, synaesthesia and the methods that should be used to verify their presence remain the subject of debate. However, there is broad consensus that synaesthesia consists of highly *specific* experiences that are *consistent* within individuals (i.e. repeated presentations of an inducer will reliably elicit the same or a very similar concurrent experience, even over long intervals of time), and which are not under voluntary control (i.e. they happen *automatically*) [1,13–15]. Note that in the examples we discuss, consistency of inducer-concurrent relations depends on, and implies, specificity.

In addition, synaesthetic experiences are nearly always reported as unidirectional; that is a grapheme may induce a colour concurrent experience, but the presentation of a coloured stimulus will typically not elicit a grapheme experience [10]. However, unidirectionality may not be universal: some evidence points to an implicit bidirectionality between an inducer and concurrent [16–18]. A less frequently noted subjective characteristic of synaesthesia is that concurrent experiences usually lack what can be called *perceptual presence* [19,20]. This means that concurrent experiences are not usually confused with, or perceived as, properties of the world. In grapheme-colour synaesthesia, for example, the concurrent colour experience is typically not confused with the actual colour of the inducer. Even though the inducer elicits an additional colour experience (e.g. red), grapheme-colour synaesthetes will still perceive the inducer as being the colour it actually is (e.g. black).

Collectively, these hallmarks distinguish synaesthesia from related phenomena, such as illusions (altered perception of a stimulus), hallucinations (similar to a concurrent experience without an inducer) and mental imagery (less or no perceptual vividness) [1,19]. Regarding this last point, there is an ongoing debate as to whether synaesthetic experiences should be viewed as distinct from vivid mental imagery [21,22].

As well as having phenomenological and behavioural hallmarks, synaesthesia also displays characteristic neurophysiological properties. In grapheme-colour synaesthesia, there is evidence for reduced phosphene thresholds on the application of transcranial magnetic stimulation (TMS) to the visual cortex, when compared to non-synaesthetes, indicating enhanced (visual) cortical excitability [23,24]. Grapheme-colour synaesthetes also show enhancements in specific visual evoked potential (VEP) components when presented with flickering checkerboard stimuli [25], suggesting enhanced modality-specific sensory–perceptual processing. (Note that these stimuli do not themselves evoke synaesthetic concurrents.) Plausibly, these neurophysiological features may reflect underlying neural mechanisms of synaesthetic experience, perhaps by enabling increased crosstalk between otherwise functionally segregated cortical regions.

The hallmarks of naturally occurring synaesthesia, the variety of its modes of expression and its widespread but variable appearance in the general population [1,26,27] highlight its relevance for investigations into the cognitive and neural basis of conscious perception. We now compare natural synaesthesia with different methods of inducing artificial synaesthesia-like experiences, with particular attention paid to the hallmarks of consistency, automaticity and (lack of) perceptual presence.

## 3. Inducing synaesthesia

### (a) Trained synaesthesia

A number of studies have explored the possibility that non-synaesthetes could acquire synaesthesia-like phenomenology through extensive associative training. These studies typically pair ‘inducer’ and ‘concurrent’ stimuli repeatedly, across a number of tasks—and then ask whether the presentation of the inducing stimulus alone elicits a synaesthesia-like ‘concurrent’ experience. Such training studies, many of which have targeted grapheme-colour synaesthesia, have reported mixed results [28–32] (see also table 1). Some behavioural effects are commonly found: for example, reaction times are typically slower for graphemes presented in colours incongruent, rather than congruent, with the trained grapheme-colour associations (a ‘synaesthetic Stroop’ effect). Importantly, though, most studies failed to elicit self-reported synaesthetic phenomenology—suggesting a failure to train synaesthesia-like *experiences*. One possible reason for this is that if natural synaesthesia depends, to some extent, on repeated exposure to combined perceptual features at key developmental stages, then one might expect training protocols in adulthood to fail unless they share the relevant characteristics of learning during development [6,47]. Two plausible characteristics are extensive training and adaptive training: *extensive* in the simple sense of time devoted to training, and *adaptive* in the sense that training tasks become progressively more challenging, both within training sessions and across the training period, serving to maintain the engagement of the participant.

**Table 1.** Summary of studies of induced synaesthesia across different methods. Each study is linked to a specific type of synaesthesia, the primary factor/method leading to the acquisition of synaesthesia-like experience and the specific hallmarks of natural synaesthesia that were successfully acquired. Hallmarks in parentheses indicate only limited evidence for that hallmark in the corresponding study.

study	type	acquisition through	hallmark
Anderson *et al*. [33]	grapheme-colour	post-hypnotic suggestion	phenomenology (behaviour)
Bor *et al*. [7]	grapheme-colour	training battery (several tasks)	phenomenology, behaviour, psychophysiology
Brang *et al*. [34]	grapheme-colour	guess-and-check	behaviour
Cohen Kadosh *et al*. [28]	digit-colour	associative learning	—
Cohen Kadosh *et al*. [8]	grapheme-colour	post-hypnotic suggestion	phenomenology, behaviour
Colizoli *et al.* [29]	letter-colour	incidental learning (reading)	phenomenology, behaviour
Colizoli *et al*. [35]	grapheme-colour	incidental learning (reading)	phenomenology, behaviour, neurophysiology
Howells [36]	sound-colour	associative learning	(phenomenology)
Jacobs *et al*. [37]	sound-phosphene	brain injury	phenomenology
Kelly [38]	sound-colour	associative learning	—
Kallio *et al*. [39]	symbol-colour	post-hypnotic suggestion	phenomenology, behaviour
Kusnir & Thut [40]	letter-colour	incidental learning (visual search)	behaviour
Meier & Rothen [30]	letter-colour	associative learning	behaviour, psychophysiology
Nair & Brang [9]	sound-phosphene	sensory deprivation	phenomenology, behaviour
Niccolai *et al.* [41]	grapheme-colour	training battery (several tasks)	behaviour
Nunn *et al*. [42]	word-colour	associative learning	behaviour
Ovalle Fresa & Rothen [43]	grapheme-colour	incidental learning (generation of associations)	behaviour
Ro *et al*. [44]	sound-tactile sensation	brain injury	phenomenology, behaviour, neurophysiology
Rothen *et al*. [32]	digit-colour	associative learning	behaviour
Rothen *et al*. [15,31]	swimming-style colour	associative learning	behaviour
Rothen *et al*. [11]	grapheme-colour	training battery (several tasks)	phenomenology, behaviour, neurophysiology
Terhune *et al*. [12]	sound-colour	drug-induced	phenomenology
Yanakieva *et al*. [45]	face-colour	drug-induced	phenomenology, behaviour
Yong *et al*. [46]	sound-phosphene	brain injury	phenomenology, neurophysiology

**Figure 1.**
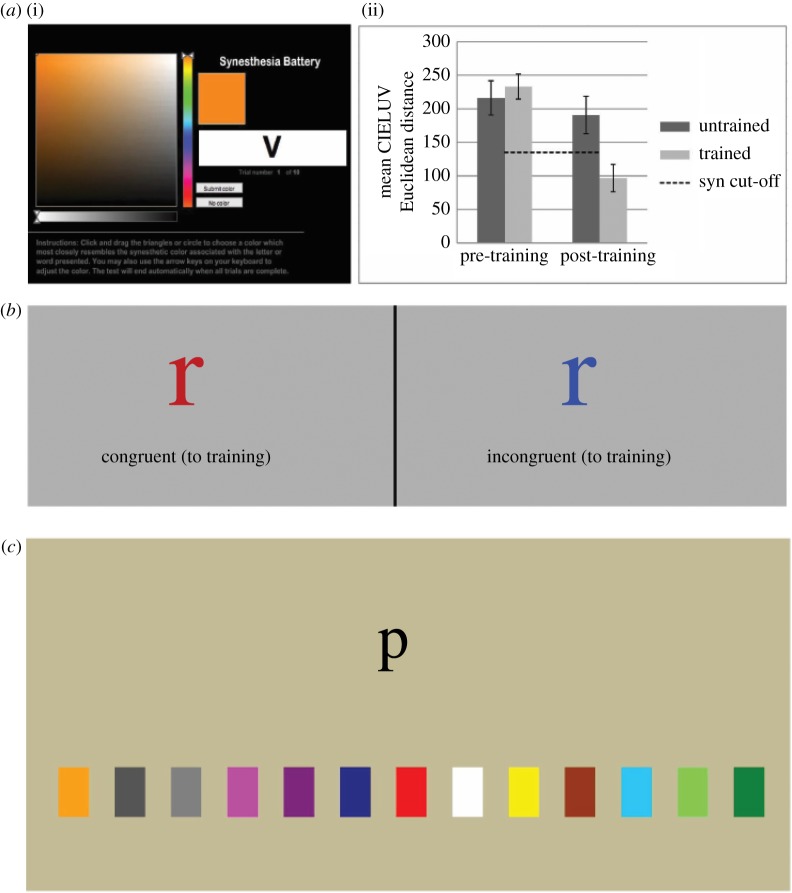
Sample training and testing tasks from Bor *et al*. [7]. *a*(i) The colour consistency test, where participants had to repeatedly choose the most appropriate colour associated with each grapheme. *a*(ii) Colour consistency scores (±s.e.), based on the CIELUV Euclidian distance algorithm [15], using the online Colour Consistency Test for the 13 trained and 13 untrained letters before and after training. A lower score reflects increased colour consistency. Values below the dashed line are usually assumed to signify genuine synaesthesia. (*b*) Examples of stimuli used in synaesthesia tests to further validate synaesthetic traits. In the Colour Naming Stroop test, participants had to name the true colour as fast as possible while ignoring the letter. In the Synaesthetic Stroop test, participants had to name the trained colour as fast as possible, ignoring the (congruent or incongruent) true colour. In both cases following training, participants were faster to respond to the congruent stimuli. (*c*) An example of one of 13 training tasks used in the study. In this case, participants saw a letter in the upper portion of the screen and had to touch the corresponding colour swatch below as fast as possible, on a touch-screen monitor.

A study from our laboratory was the first to use such an extensive and adaptive regime in an attempt to more closely mirror the supposed real-life development of synaesthesia [7]. Basic features of the study are summarized in figure 1. Using the gold-standard colour-consistency test for the behavioural diagnosis of synaesthesia ([14,15] www.synesthete.org, now hosted by the University of Sussex), we found that only trained graphemes demonstrated levels of consistency indicative of synaesthetic experience [15] (figure 1*a*(i,ii)). Further tests, such as the synaesthetic equivalent of the Stroop test [30,48], also demonstrated synaesthesia-like behaviour for the trained letters (figure 1*b*).

Critically, this study was also successful at generating self-reported synaesthetic phenomenology. Out of 14 participants, only 2 reported no synaesthetic phenomenology. The nature of this reported phenomenology varied across participants, including examples of both projector-type reports (in which concurrents are experienced as being outside of bodily space, ‘projected’ into the world) and associator-type reports (in which concurrents are experienced within an internal mental space without any distinct spatiality; see [49] for more on this distinction). In our study, an example of a projector-like report was as follows: ‘When I was walking into campus I glanced at the University of Sussex sign and the letters were coloured’ (according to the trained associations). By contrast, an associator-like report was: ‘When I look at the letter ‘p’ I know the colour pink goes with it, it's like inside my head is pink’.

A second training study, also from our laboratory, addressed two additional neurophysiological hallmarks associated with grapheme-colour synaesthesia [11]. This second study also contained additional control groups to exclude the possibility that synaesthetic phenomenology could result simply from extensive associative training alone. This study consisted of three participant groups: (i) experimental, (ii) active control and (iii) passive control, but was otherwise very similar to the earlier study.

**Figure 2.**
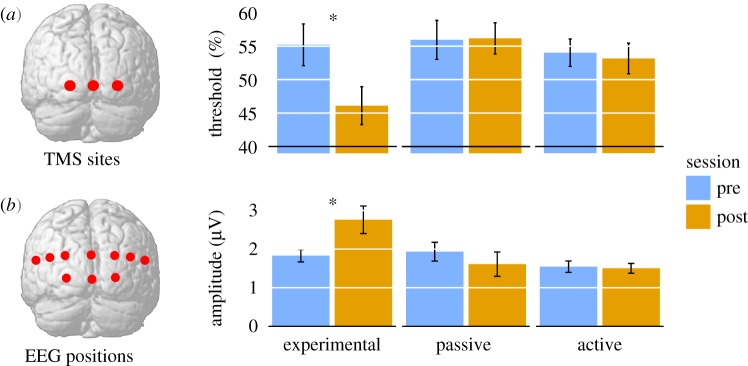
Selected results from Rothen *et al*. [11]. (*a*) TMS-evoked phosphene test. Only the active group showed a post-training reduction in phosphene thresholds. (*b*) VEP amplitude of C3 component. Only the active group showed a post-training increase in VEPs elicited by checkerboard stimuli. Figure adapted from Rothen *et al*. [11].

Replicating the results of Bor *et al*. [7], Rothen *et al*. [11] found performance on the consistency test for the trained letter-colour pairs that passed the threshold for natural synaesthesia. In addition, all participants in the experimental group (18/18) also demonstrated letter-specific behavioural effects (e.g. consistency and synaesthetic Stroop) and phenomenology suggestive of natural synaesthesia (for similar effects on consistency, see [43]). Extending the previous study, these effects were found only for the experimental group—and not for either of the control groups. In addition, for the neurophysiological assessments, only the experimental group demonstrated a post-training reduction in TMS phosphene threshold and an enhanced EEG VEP response (figure 2). These effects were of a similar effect size to those reported when comparing natural synaesthetes with non-synaesthetic controls [24,25].

The original training studies did not report specific data regarding the phenomenological hallmark of ‘perceptual presence’—the characteristic of natural synaesthesia whereby concurrents are not experienced as being ‘part of the world’. However, verbal free reports from both studies suggest that, as with natural synaesthesia, trained synaesthetic experience also lacked perceptual presence. For the present review, we conducted an additional content analysis of the phenomenological reports from the second study [11,50] with a focus on perceptual presence. This analysis indicates that all participants experienced dual, distinct percepts for the inducer and concurrent (for instance, ‘r’ being both black on the page and inducing redness either near that location or in their mind's eye). In addition, no participants were confused as to which was the veridical (i.e. part-of-the-world) percept. A number of spontaneous reports highlighted the lack of perceptual presence as motivation for rating trained concurrent experiences as ‘weaker’ than a colour association for a real-world object. Here is one example: ‘I would say that they feel roughly the same, but it might sound stupid, but like tomato is more of a real colour, but when I think of the green (trained colour) I think of it on a computer screen and it's a sort of not a real thing, so it doesn't feel as natural. When you think of a tomato you think about how the light hits something, that shape, and that's part of the colour’. This single quote nicely illustrates a lack of perceptual presence for trained synaesthesia, akin to natural synaesthesia. More comprehensive analyses of these reports will be presented separately [50]; for now, they remain suggestive rather than conclusive.

Altogether, the results from these training studies provide compelling evidence that synaesthesia-like experiences can be acquired by non-synaesthetic individuals through extensive, adaptive and targeted cognitive associative training. Such training can lead to a coordinated suite of behavioural, phenomenological and neurophysiological changes, akin to natural synaesthesia. We now compare these findings with other methods for artificially inducing synaesthesia-like experiences and behaviour.

### (b) Pharmacologically induced synaesthesia

The use of psychedelic drugs, such as psilocybin, LSD and mescaline, has a long history of reportedly inducing synaesthesia-like experiences, such as the cross-modal induction of colour experiences via auditory tones [51,52]. However, due to the methodological limitations of these studies, including lack of placebo controls and a failure to assess key hallmarks of synaesthesia within these experiences, it has been difficult to draw firm conclusions about the validity of these claims (for reviews, see [52,53]). For example, drug-induced synaesthesia-like experiences seem to lack consistency, do not occur automatically and can be influenced by the current state of the individual, whereas a (natural) synaesthetic concurrent is consistent, automatic and unaltered by an individual's current state of mind [53].

One recent study examined synaesthesia-like experiences induced by LSD using more of a methodologically sophisticated approach [12]. However, this study found only weak spontaneous synaesthesia-like experiences, which lacked inducer-specificity. In addition, no differences in the consistency of concurrent experiences were found between LSD and placebo conditions for grapheme-colour or sound-colour associations. This study concluded that, at best, LSD produced only weak synaesthesia-like experiences, which lacked the hallmarks of natural synaesthesia. By contrast, recently a case was reported in which multiple forms of synaesthesia were acquired after a single very high dose of the psychedelic drug 2-CB [45]. These experiences exhibited inducer-concurrent consistency for week-colour and instrument-colour synaesthesia [14] and automaticity for face-colour synaesthesia (assessed via a priming task).

The perceptual presence—or lack thereof—of pharmacologically induced synaesthesia-like experiences has not been directly assessed. However, it seems plausible that at least some of these experiences, as with psychedelic experiences more generally, show some continuity with other perceptions and so admit the quality of perceptual presence [19]. Pharmacologically induced hallucinatory experience can vary enormously within and across individuals [51,54,55], and further research is needed to fully characterize perceptual presence in these cases.

In summary, while there is ongoing debate about whether certain pharmacologically induced experiences should be described as synaesthesia-like [21,52,53], currently it appears that these experiences represent a more dynamic and flexible phenomenon, having only superficial similarities to natural synaesthesia.

### (c) Hypnotically induced synaesthesia

Hypnosis is now well established as a means of altering the phenomenological properties of participants' subjective experience [56,57]. This ability has now been leveraged to induce abnormal cross-modal experiences in non-synaesthetes that bear similarities to natural synaesthesia [8,39].

In a seminal study, Cohen Kadosh *et al*. [8] hypnotized four highly suggestible participants to associate digits with specific colours (1—red, 2—yellow, etc.). Under post-hypnotic suggestion, various tests were administered including a digit detection task in which participants were asked to detect the presence or absence of a digit presented on differing colour backgrounds. Cohen Kadosh *et al*. [8] found that during post-hypnotic induction, when a black digit was presented on a background that was congruent to the number–colour association (e.g. 1 was presented on a red background), participants made substantially more errors compared to incongruent presentations, with no such effect in control groups. This finding is in line with the ‘automaticity’ hallmark of natural grapheme-colour synaesthetes, who are also more prone to making errors when detecting the presence of a digit in the congruent condition [58]. In addition, subjective reports following post-hypnotic induction were found to resemble those of grapheme-colour synaesthetes, with these experiences demonstrating some degree of consistency. A limitation here is that consistency was assessed using subjective reports rather than the gold-standard behavioural test, as used in the training studies discussed above. Nevertheless, this hypnosis study provides compelling evidence that non-synaesthetes can be hypnotically induced to exhibit phenomenologically comparable concurrent experiences, as well as a behavioural marker (automaticity) associated with natural synaesthesia.

A more recent study attempted to induce form-colour synaesthesia using hypnotic suggestion [39]. Four highly suggestible participants were given the hypnotic induction that symbols within an array (circles, crosses or squares) would always display a certain colour, even though they were presented in differing colours. Kallio *et al*. found that under post-hypnotic suggestion, three out of the four participants displayed synaesthesia-like phenomenology, with two of these participants reporting projector-like concurrent experiences. An interesting aspect of this study is that participants described the veridical colour of the symbol as being ‘replaced’ by the colour of the concurrent, such that they perceived only the concurrent colour. Kallio *et al*. put it this way: ‘Their subjective experience appeared very similar to a projector-type synaesthete, with the key difference that they reported the object as having the suggested colour’ [39, p. 7]. This marks a difference with respect to natural synaesthesia, suggesting that hypnotically induced synaesthesia-like experiences do indeed have some perceptual presence—since they would seem to be confusable and continuous with other perceptions. Indeed, Kallio *et al.* conclude that while hypnotic suggestion can create a condition that displays some functional similarities to synaesthesia, it also displays clear differences [39]. We note that the findings of Cohen Kadosh *et al.* [8] are also interpretable in this way—failure to detect a (black) digit when presented on a background congruent with the (induced) colour is compatible with the idea of the induced concurrent perceptually ‘replacing’ the inducer.

Another hypnosis study, which used a larger sample of highly suggestible participants (14), reported only limited similarities between natural and hypnotically induced synaesthesia [33]. Following post-hypnotic suggestion, which aimed to induce the projector variant of grapheme-colour synaesthesia (e.g. perceive the number 5 as red and the number 2 as green), Anderson *et al.* [33] presented participants with embedded figures that consisted of shapes (e.g. squares or triangles) of number 2s embedded in an array of 5s. In natural synaesthesia, the associated concurrent experience has been reported to aid synaesthetes in locating and identifying the embedded shape in this task [3,59]—though these effects fall short of full perceptual ‘pop out’. By contrast, Anderson *et al*. [33] found that, following post-hypnotic induction, participants lacked any behavioural advantage compared to controls, even when they restricted their analysis to the participants who responded most strongly to the hypnotic suggestion. However, they did find that participants' phenomenological reports of hypnotically induced colour experiences displayed similarities to those of natural synaesthesia. The authors conclude that while phenomenological similarities exist, the lack of behavioural improvement observed following induction suggests that hypnotically induced colour experiences are not equivalent to the experiences of natural synaesthesia.

Altogether, the studies reviewed here indicate that post-hypnotic induction is able to rapidly induce additional perceptual experiences that bear some similarities to natural synaesthesia, for example, automaticity and some degree of consistency—though perceptual presence may again represent a difference, and requires further study. Despite these similarities, the low sample sizes of some of these studies [8,39] and an inability of others to replicate key behavioural findings [33] preclude firm conclusions about the equivalence of hypnotically induced and natural synaesthetic experience.

### (d) Synaesthesia as the result of sensory deprivation

Synaesthesia due to sensory deprivation has been studied primarily in the context of patients who have experienced profound visual loss, such as deafferentation of the visual system [37,46,60]. For example, Jacobs *et al*. [37] described nine patients who suffered a visual loss due to lesions of the optic nerve. All these patients experienced photisms (simple flashes or kaleidoscopic patterns) in response to sound within as little as 1–3 days following the visual loss. To the extent that these situations can be described in terms of inducers (sounds) and concurrents (photisms), there is little evidence for consistency in the inducer-concurrent coupling: the same inducer evoked different concurrent experiences at different times [37]. In addition, the phenomenologically simple nature of these experiences makes them difficult to interpret in terms of consistency. If multiple inducers elicited a similar ‘simple’ concurrent experience (flash of light), it is possible that they could be misinterpreted as displaying a stable mapping.

Another line of investigation has studied the effects of sensory deprivation by blindfolding sighted participants for short periods of time. Nair & Brang [9] report that with as little as 5 min of visual deprivation, they could automatically elicit visual experiences (flashes or patterns) in 57.1% (*n* = 77) of sighted participants using auditory stimulation. However, these experiences seem to lack consistency, with the same tone either not eliciting a concurrent experience or eliciting a different visual experience at different times. It is possible that, similar to training-induced synaesthesia, the consistency of auditory-visual experiences of blindfolded sighted participants could develop over time through repeated pairings.

Summarizing, acquired and induced forms of sensory deprivation may induce some synaesthesia-like cross-modal experiences; however, the similarities with natural synaesthesia are likely only superficial. Nonetheless, the fast onset and transient nature of induced cross-modal experiences in these cases underline the malleability of perceptual experience. Unfortunately, due to participants either experiencing a visual loss or being blindfolded, the issue of perceptual presence cannot be readily examined in these cases.

### (e) Synaesthesia due to brain injury

In rare situations, synaesthesia-like experiences have also been reported following specific forms of brain damage [44]. It is commonly assumed that in these cases the synaesthesia-like experiences are the result of functional and structural reorganization (neuroplasticity) of the brain following injury. In an intriguing case study, following a focal lesion to the ventrolateral nucleus of the thalamus, a patient found that auditory stimuli now produced tactile percepts [44]. Based on repeated testing over a 3-year period and MRI and diffusion tensor imaging investigations, the authors concluded that this specific instance of acquired synaesthesia was the result of abnormal connectivity between the right ventromedial thalamus and the cortex. They suggest that reduced thalamo-cortical interactions may have strengthened the connectivity between auditory and somatosensory cortices, which led to the experience of sound-touch synaesthesia in this patient.

In further analyses, they tested the consistency of these cross-modal experiences, finding that the majority (91%) of the repeated sounds produced consistent tactile percepts. However, the authors note that, in addition to sound-touch synaesthesia being a rare subtype of developmental synaesthesia [61], the acquired concurrent somatosensory experiences of this patient were relatively simple in nature (pressure or tingling) compared to genuine synaesthetic experiences, perhaps leading to an overestimation of consistency. Thus, while this single case study displays some of the hallmarks of natural synaesthesia, it also differs in many ways. Again, in this case, due to the tactile nature of the concurrent experience, the issue of perceptual presence cannot be readily examined.

## 4. Discussion

In this review, we have examined a range of examples of artificially induced synaesthesia. By evaluating these synaesthesia-like experiences against widely agreed upon hallmarks of natural synaesthesia, we conclude that certain aspects of synaesthesia can be induced in non-synaesthetes via a wide variety of approaches. Among these approaches, alterations in phenomenology induced via sensory deprivation [9,62], the ingestion of psychoactive substances [12,51], as well as acquired forms of synaesthesia, for instance, following brain damage [44,46], bear only superficial similarities to natural synaesthesia. By contrast, post-hypnotic induction was able (in some cases) to induce additional perceptual experiences showing greater resemblance to natural synaesthesia [8,39]. Extensive, adaptive and targeted cognitive training provides the most compelling evidence, thus far, that synaesthesia-like experiences can be acquired by non-synaesthetic individuals [7,11].

Table 1 provides a summary, listing 24 separate studies. These are organized by the type of synaesthesia-like experience produced, its method of induction and the hallmarks of natural synaesthesia reproduced. Altogether, these studies collectively reveal a surprising potential for alteration of perceptual experience, even in adults, with some instances of this altered experience sharing key characteristics of natural synaesthesia. This was especially striking for recent extensive and adaptive training studies [7,11] but may also apply to some hypnotically induced synaesthesia-like experiences as well [8,39].

We have focused on three hallmarks of natural synaesthesia: *consistency*, *automaticity* and (lack of) *perceptual presence*. Only the training paradigms have so-far been able to establish consistency to an extent comparable with natural synaesthesia. These training paradigms also demonstrated automaticity using adapted versions of the Stroop test. Post-hypnotic suggestion is able to induce phenomenologically comparable concurrent experiences to natural synaesthesia, with some degree of automaticity [8,39]. However, an inability to replicate other behavioural effects [33], combined with weak evidence of consistency, preclude firm conclusions about the equivalence of hypnotically induced and natural synaesthetic experiences.

Drug-induced synaesthesia-like experiences appear to lack inducer-specificity, consistency and do not occur automatically in response to an inducer [12,53]. Similarly, acquired and induced forms of synaesthesia as the result of sensory deprivation appear to lack consistency, and the phenomenologically simple nature of these experiences suggest only superficial similarities to natural synaesthesia. Finally, rare cases of synaesthesia due to brain injury are capable of inducing specific forms of synaesthesia (auditory-tactile) [44], which display high levels of consistency. However, the resulting tactile experiences are relatively simple in nature (pressure or tingling) compared to natural synaesthetic experiences, suggesting that these experiences differ in many respects to natural synaesthesia.

When it comes to the often-overlooked subjective characteristic of perceptual presence [19], training-induced synaesthesia-like experiences also appear to lack perceptual presence, as in natural synaesthesia. However, this does not by itself indicate phenomenological equivalence between training-induced and natural synaesthesia. Ongoing analysis will shed further light on this question [50]. It remains an open question if hypnotically induced synaesthesia-like experiences also lack perceptual presence. However, indirect evidence showing that the veridical colour of the inducing stimuli is replaced or occluded by the colour of the concurrent experience suggests that, unlike natural synaesthesia, these experiences do have some perceptual presence [8,39]. Perceptual presence may feature even more strongly for drug-induced perceptual experiences [19]. We cannot speak to the perceptual presence of experiences during sensory deprivation or sensory substitution as, in both cases, participants’ vision was obscured. Further exploration of the phenomenology of induced and genuine synaesthetic experiences using extensions of the theoretical frameworks of sensorimotor theory [63] and predictive processing [64,65] has great potential to provide insights into the unique phenomenological characteristics of synaesthesia, as well as other experiential states [19].

The striking alterations in perceptual phenomenology observed in many of the reviewed cases of induced synaesthesia, combined with the rapid onset of some of these perceptual changes, together highlight the potential plasticity of perceptual phenomenology, even in adults. Collectively, these results support the notion that cortical perceptual representations in adults are not fixed, but rather, are dynamic and continuously altered by experience. Considering the training paradigms, dramatic alterations in perceptual phenomenology occurred within a relatively brief timeframe (less than 24 h total training), despite a life-long prior history of no additional perceptual experiences for letter stimuli. Even more notable are the compelling reports of induced synaesthesia as the result of post-hypnotic suggestion and sensory deprivation, which demonstrate that synaesthesia-like phenomenology can be brought about within a much shorter timeframe, in some cases in as little as 5 min [8,9,39].

Such rapid changes in perceptual phenomenology are difficult to accommodate within development models of natural synaesthesia, such as the cross-activation model. Such models propose that synaesthesia arises over months or years through a greater-than-normal structural connectivity between cortical representations of inducers and concurrents [66,67]. Instead, the fast onset of induced synaesthesia-like phenomenology—in both the training and hypnosis paradigms—is more compatible with the disinhibited-feedback model [68–70], which posits that synaesthesia occurs through the same neural connections as in a ‘non-synaesthetic’ brain, but it is the alterations in functionally driven disinhibited feedback between or within brain areas that generate concurrent experiences (for a related perspective, see Smilek *et al*. [58]).

Additional though indirect support for the disinhibited-feedback model is provided by the increased cortical excitability and enhanced visual perceptual processing found in our second training study [11]. A decrease in normal inhibitory processes due to training could plausibly increase cortical excitability, therefore reducing phosphene thresholds. Interestingly, individual variations in cortical excitability have been shown to underlie some of the phenomenological differences observed in grapheme-colour synaesthesia. For example, projector synaesthetes display greater cortical excitability (lower phosphene threshold) than associator synaesthetes, with both exhibiting lower thresholds compared to controls [23]. These differences are plausibly related to the enhanced visual perceptual processing found in grapheme-colour synaesthetes [25]. Some of the other forms of induced synaesthesia reviewed here also support this line of reasoning. The synaesthesia-like experiences induced by sensory deprivation [9,62], psychedelic drugs [71,72] and brain injury [71], have all been theorized to occur due to enhanced cortical excitability. Therefore, we speculate that for some forms of induced synaesthesia, the common mechanism that underlies the alterations in perceptual experiences may be an increase in cortical excitability within perceptual brain regions.

The associative nature of synaesthesia has led many to speculate that a learning component must be involved in the development of specific synaesthetic associations, especially when the inducers are cultural artefacts, as it is the case in grapheme-colour synaesthesia [5,6,10,43]. In line with this claim, the induced forms of synaesthesia reviewed here cast doubt on claims that genuine synaesthetic phenomenology can only occur in a rare subset of the population that exhibit a genetic predisposition [73,74]. Instead, the success of these differing approaches, particularly extensive training, in inducing synaesthesia-like experiences points to natural synaesthesia having a substantial dependence on learning and prior experience [6,10,75], potentially founded on conceptual associations that aid learning processes [47,76]. In this view, it is not surprising that the results of cognitive-perceptual training displayed a greater number of the hallmarks of natural synaesthesia (phenomenological, behavioural and neural) compared to other induction methods, as the extensive associative learning performed by participants in both studies may more closely resemble the likely developmental trajectory of natural synaesthesia [7,11]. For this reason, it will be interesting to investigate in future studies whether training-induced synaesthesia is associated with cognitive benefits found in natural synaesthesia, such as an enhanced memory (for a review, see [2]). A first training study, which examined if grapheme-colour associations are based on the consolidation of environmental contingencies, provides preliminary evidence that this may be the case [43].

## 5. Summary

Synaesthesia provides a fascinating window on conscious perception, providing opportunities to bridge from genetics [4], to neurobiology, to behaviour and finally to phenomenology. Reviewing several forms of artificially induced synaesthesia—with a focus on the consistency and automaticity of the resulting synaesthesia-like experiences—we conclude that a variety of approaches can be used to induce phenomenological, behavioural and, in some cases, neural hallmarks similar to those found in natural synaesthesia. The subjective characteristic of ‘perceptual presence’ stands out as an important phenomenological benchmark for future studies of artificially induced synaesthesia-like experience. Collectively, the rapid onset and dramatic changes in phenomenology observed in induced forms of synaesthesia highlight the potential for perceptual plasticity even in adulthood and underlines the likely importance of lifetime experience and learning in the development of natural synaesthesia.

## Data accessibility

This article has no additional data.
